# The usability and applicability of knowledge translation theories, models, and frameworks for research in the context of a national health service

**DOI:** 10.1186/s12961-021-00747-5

**Published:** 2021-07-26

**Authors:** Virginia Minogue, Karen Matvienko-Sikar, Catherine Hayes, Mary Morrissey, Gregory Gorman, Ana Terres

**Affiliations:** 1 Research and Development, Strategy and Research, Health Service Executive, Dublin 8, Ireland; 2 School of Public Health, University College Cork, College Road, Cork, Ireland; 3 School of Medicine, Public Health and Primary Care, Trinity College, College Green, Dublin, Ireland; 4 Health Intelligence, Health Service Executive, Dublin, Ireland; 5 Research and Evidence, Strategy and Research, Health Service Executive, Dublin 8, Ireland

**Keywords:** Knowledge translation theories, models, and frameworks, Knowledge dissemination, Health services research, Research culture, T-CaST

## Abstract

**Background:**

Translating research findings into service improvements for patients and/or policy changes is a key challenge for health service organizations. The Health Service Executive (HSE) in Ireland launched the Action Plan for Health Research 2019–2029, as reported by Terrés (HSE, Dublin, 2019), one of the goals of which is to maximize the impact of the research that takes place within the service to achieve improvements in patient care, services, or policy change.

The purpose of this research is to review the literature on knowledge translation theories, models, and frameworks (TMFs) and to assess the suitability of the TMFs for HSE use, selecting one or more for this purpose. The aim is to produce guidance for HSE researchers and other health services staff, validate the usability of the framework(s) with researchers, and review and implement the guidance. It was hoped that identifying a suitable methodology would provide the means to increase the uptake and application of research findings, and reduce research wastage. This paper reports on the first part of the study: the review, assessment, and selection of knowledge translation TMFs for a national health service.

**Methods:**

An interdisciplinary working group of academic experts in implementation science, research wastage, and knowledge translation, along with key representatives from research funders (Health Research Board) and HSE personnel with expertise in quality improvement and research management, undertook a three-stage review and selection process to identify a knowledge translation TMF that would be suitable and usable for HSE purposes. The process included a literature review, consensus exercise, and a final consensus workshop. The review group adopted the Theory Comparison and Selection Tool (T-CaST) developed by Birken et al. (Implement Sci 13: 143, 2018) to review knowledge translation theories, models, and frameworks.

**Results:**

From 247 knowledge translation TMFs initially identified, the first stage of the review identified 18 that met the criteria of validity, applicability, relevance, usability, and ability to be operationalized in the local context. A further review by a subgroup of the working group reduced this number to 11. A whole-group review selected six of these to be reviewed at a facilitated consensus workshop, which identified three that were suitable and applicable for HSE use. These were able to be mapped onto the four components of the HSE knowledge translation process: knowledge creation, knowledge into action, transfer and exchange of knowledge, and implementation and sustainability.

**Conclusion:**

The multiplicity of knowledge translation TMFs presents a challenge for health service researchers in making decisions about the appropriate methods for disseminating their research. Building a culture that uses research knowledge and evidence is important for organizations seeking to maximize the benefits from research. Supporting researchers with guidance on how to disseminate and translate their research can increase the uptake and application of research findings.

The use of robust selection criteria enabled the HSE to select relevant TMFs and develop a process for increasing the dissemination and translation of research knowledge. The guidance developed to inform and educate researchers and knowledge users is expected to increase organizational capacity to promote a culture of research knowledge and evidence use within the HSE.

## Background

A high proportion of health services research is wasted [3], with failure to translate research findings into research policy and practice often being a significant contributor to waste [4]. The Irish Health Service Executive (HSE) Research and Development office launched its Action Plan for Health Research 2019-2029 in December 2019 [1]. The HSE wanted to enhance a culture where research was valued and benefited the health service and service users. It also has as one of its goals that its research could lead to improvements in patient care, services, or policy change. The Action Plan for Health Research [1] includes, as one of its six aims, to:

*Implement institutional measures to facilitate the translation of research into policy and practice, and increase dissemination of knowledge.*

In order to identify and implement best practice to facilitate knowledge translation (KT), the central HSE Research and Development office formed the Research Translation, Dissemination, and Impact working group made up of 13 people with relevant experience and expertise in the topic. The members of the group were drawn from the HSE, three academic institutions, and the Health Research Board, and brought experience in public health, evidence and improvement, research management, research funding and awards, health library and knowledge services, and clinical and health psychology. The purpose of the working group was to produce guidance for HSE researchers on how to carry out dissemination and translation of research to achieve an impact. The group was also tasked with identifying a suitable methodology for the HSE to increase the uptake and application of research findings and enhance their impact,1 and to reduce research wastage. This is intended to increase organizational capacity to promote innovation and a culture of evidence.

A range of KT theories, models, and frameworks (TMFs) have been published to date that might be relevant to health services research, as evidenced by recent reviews in the area [5, 6]. However, many of these TMFs have not been fully tested or lack evidence of their effectiveness [6], particularly within health organizations. Moreover, few national/public health systems have developed a strategy for translating and disseminating research into practice and policy. One example of a system that improved organization-wide KT by focusing on contextual factors within unique health departments is the Canadian Public Health Service [7]. Various research funders, such as the Canadian Institutes of Health Research and the United Kingdom National Institute for Health Research, have sought to increase the impact of their funded research and are prioritizing KT activities; however, there is no clear and consistent approach to engaging users of research through integrated knowledge translation (IKT) [8]. Although research funding support for KT activity is to be welcomed, it is clear that for research to have an impact, health partners and other stakeholders must be engaged and in a position to implement research and put processes in place to do so. The culture within health service organizations may be a barrier to or facilitator of successful implementation, for example through their support, or lack of support, for research and innovation, and the quality of their relationships with academia [9]. Studies, including a survey of HSE researchers [10–12], identified a range of structural and organizational barriers to and facilitators of dissemination of research to appropriate audiences. The barriers were primarily time constraints, lack of research support from the organization, passive methods of dissemination, and lack of researcher links with policy-makers to enable impact. The factors that could facilitate the dissemination and translation of research were as follows:

- Thinking about dissemination and KT at the research planning stage
- Time and resources
- An organizational culture that values and is committed to research
- Leadership and organizational support
- Building partnerships and capacity
- Having a relevant research question
- Stakeholder engagement
- Identifying clear messages from the research and targeting information to specific audiences.

The multiplicity of KT TMFs is also likely to be a deterrent to health organizations’ ability to optimize the use of research, by obfuscating clear and effective KT procedures. Context has been referenced as an important component by a number of TMFs, including the organizational context, the need for organizational buy-in, and an organizational culture that facilitates KT and is open to change [5, 7, 9, 15, 16]. The ability of researchers to mobilize their research into policy and practice and to achieve maximum impact must be supported by the health organizations in which they work. Examples of where this has happened are illustrated in the literature and demonstrate the importance of organizations engaging in building capacity and understanding what works and in what setting [7, 17]. Understanding how policy-makers, managers, and clinicians receive and use knowledge and evidence can help knowledge creators recognize how KT can be translated for best effect [18]. Timely, continuous, and targeted KT is also fundamental to its success [7, 17]. A decision support tool that assists researchers in selecting the most appropriate method of KT and informs organizational guidance would therefore be beneficial [6]. This can help ensure the translation of health services research in an evidence-based, continuous, timely, and targeted way, supported by the health organizations in which researchers work. Therefore, the aim of this study was to develop guidance for HSE researchers and other health services staff on the use of selected TMFs, to assist them in increasing the dissemination of their research and, hence, enhancing its impact.

## Methodology

**Fig. 1:**
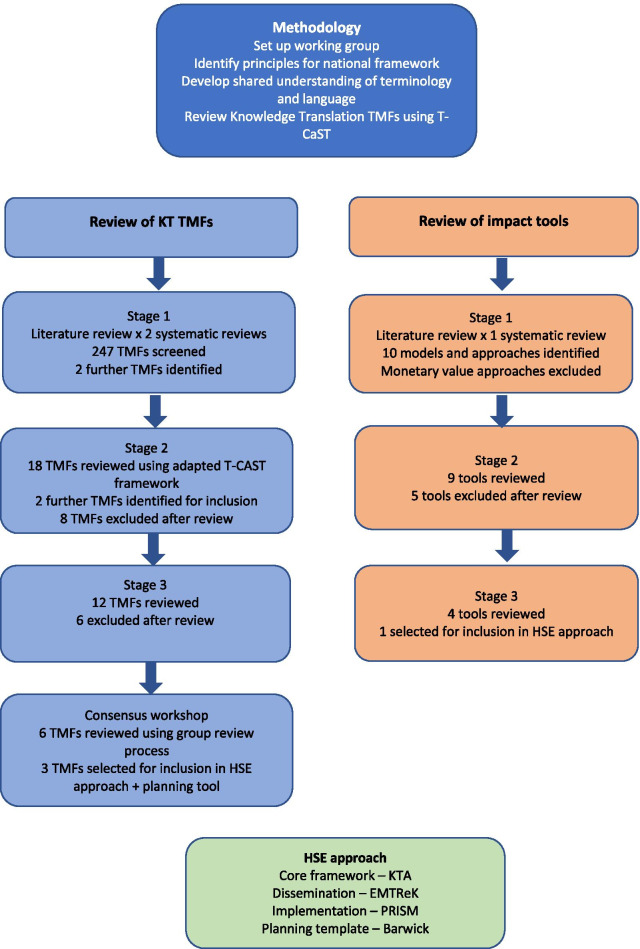
Developing an HSE approach to KT, dissemination, and impact

The Research Translation, Dissemination, and Impact working group undertook a review and selection process in order to identify a KT TMF that would be suitable and usable for HSE purposes (see Fig. 1). The process took place over a period of four months (August to December 2019) in the form of three stages of review outlined below. Having agreed upon the terms of reference, a set of principles, shared language, and terminology to be used in developing the guidance was developed during consensus discussions among the working group.

To facilitate the review process, a small subgroup consisting of four members of the working group was set up (the two HSE project leads and two academics with specific expertise in the use of KT frameworks). The subgroup set the parameters for the review, selected and applied relevant criteria from the review tool, the Theory Comparison and Selection Tool (T-CaST) [2], and undertook the first three rounds of the review, reporting to the working group at their monthly meetings. The consensus exercise and final review was undertaken by the whole working group. Additional resources for the initial review and identification of the literature were provided by one of the authors (GG) through a member of the working group. The T-CaST was selected because it is a tried and tested tool for selecting TMFs, includes a scoring system that can be replicated, and is flexible in enabling the user to select the criteria relevant to their project [2].

Prior to its utilization by the subgroup, the T-CaST was piloted and tested by two members of the project working group on two TMFs. They reported their findings to the project working group, who were satisfied that the tool was reliable and valid for use. While there has been no formal assessment of the reliability of the T-Cast to date, the approach to the development of the T-CaST [2] reflects a comprehensive approach to tool development that includes relevant stakeholders and processes to maximize validity. Further, the validity of T-CaST is recognized, and it has been recommended for use as an appropriate tool [20, 25].

The stages of the review and selection process were as follows:

### Stage 1: Review to identify and evaluate relevant TMFs and initial screening process

An initial unstructured review of the literature identified two recently published systematic reviews [5, 6] which addressed the study aim of identifying existing TMFs. To avoid unnecessary duplication and potential research waste from conducting an additional review on the same topic, these two systematic reviews were used as the basis for identification of TMFs in the current study. Details of the TMFs identified in these reviews were extracted and recorded in an Excel spreadsheet, and full texts of related TMF papers were subsequently obtained for review by the project team. Throughout this process, the review team remained open to the potential inclusion of additional TMFs for review that may not have been included and/or were published subsequent to the reviews; this was done to ensure inclusivity of TMFs.

The subgroup set the criteria for the initial selection of TMFs based on the categories identified in the T-CaST (usability, testability, applicability, acceptability)^1^ and the context—that is, the specific needs of the HSE for a TMF to be health service-based, usable, and able to be operationalized in the Irish health system. Selected TMFs should be:

- Validated
- Applicable to health services and show the extent of their use in health services
- Relevant to KT, dissemination, and impact
- Feasible and usable
- Able to be operationalized in an Irish health context

All identified TMFs were independently rated by at least two reviewers according to the criteria above and using the T-CaST criteria to determine eligibility for inclusion in stage 1.

### Stage 2: Screening of TMFs

To be eligible for the next stage of screening, TMFs identified in stage 1 were screened according to the following criteria, based on the T-CaST tool:

- TMF is for health service use
- Relevant to knowledge, dissemination, and impact
- Usability and acceptability (TMF that can be understood, applied, and operationalized in the Irish Health Service)
- Validated
- Applicability (addresses a relevant analytic level; generalizable to relevant populations and/or conditions) (See Table 1).

**Table 1:** Stage 2: TMF screening criteria based on the T-CaST framework

Criteria	Rating (yes/no)	Additional comments
TMF is for health service use (include consideration of the extent of use)		
Relevant to knowledge, dissemination, and impact (with impact as less priority in this context)		
Usability and acceptability: TMF that can be understood, applied, and operationalized in the Irish Health Service		
Validated:		
Applicability: addresses a relevant analytic level; generalizable to relevant populations and/or conditions		
Final decision to include or exclude:

The process was the same as the initial screening; reviewers were asked to provide a yes/no rating for each of the criteria, an overall assessment of include or exclude, and a rationale for their assessment. An initial pilot screening of three TMFs was conducted by five reviewers (GG, CH, MM, VM, KM-S). This was done to ensure the appropriateness of the above screening processes and criteria and to assess inter-rater reliability.

### Stage 3: Consensus review and workshop to identify HSE TMFs using the T-CaST tool and discussion

Prior to a face-to-face consensus workshop, all working group members were asked to review the remaining TMFs identified at stage 2 using a prepared review form (see Table 2). This form was also based on T-CaST and involved scoring each TMF for its acceptability, usability, relevance, and applicability to HSE and its validity, using the T-CaST scoring system (0 = poor fit; 1 = moderate fit; 2 = good fit; 3 = very good fit) [2]. This process adopted a more detailed approach to reviewing and scoring TMFs than in previous stages in order to prioritize TMFs to bring forward to the final consensus process. In order to arrive at a definitive list of TMFs for review at the consensus event, the data were reviewed in five different ways as follows:

1. The total score for each TMF across all raters
2. The average score for each TMF across all raters
3. Identifying the five TMFs with the highest total score for each rater
4. Identifying the TMFs given a score of 20 or more by each rater
5. From 3 and 4 above, identifying which TMFs were most frequently given the highest scores by the raters.

**Table 2:** Stage 3: TMF consensus review criteria based on T-CaST framework

Criteria	Rating (0—3)	Additional comments
Acceptability: TMF is for health service use (include consideration of the extent of use)		
Relevance: relevant to knowledge, dissemination and impact (with impact as less priority in this context)		
Usability and acceptability: TMF that can be understood, applied and operationalized in the Irish Health Service		
Usability and acceptability: the TMF has a clear and useful figure depicting included constructs and relationships among them		
Usability and acceptability: the TMF provides a step by step approach for applying it		
Usability and acceptability: the TMF provides methods for promoting implementation in practice		
Validated: the TMF is supported by empirical data		
Applicability: addresses a relevant analytic level; generalizable to relevant populations and/or conditions		
Final decision to include or exclude: 0 = poor fit; 1 = moderate fit; 2 = good fit; 3 = very good fit		

A rating of “0” or “1” for any criteria means the final decision should be exclude but there may be exceptions to this—if so, please specify rationale in “additional comments”

### Consensus workshop

All members of the project working group were invited to a day-long workshop. Nine people attended the event, which was led by a facilitator (an HSE national lead for well-being programmes) who was not involved in the project. The attendees included the two HSE project leads, three members of the HSE research and evidence team, two academics, and a manager from the national health service library. The facilitator worked with the group to identify the outcomes from the workshop and the key features that HSE would wish to find in a tool. These were discussed in a plenary session and agreed as follows:

- Health research moved into practical use
- Best available evidence on health
- More clarity for busy researchers/users
- People using a practical tool to support them
- Demonstrate commitment to research impact
- Better commissioning process
- Reduce research waste—explore what has been done already, consider existing evidence base.

The criteria for assessing the TMFs was similarly discussed and agreed (see Table 3).

**Table 3:** Criteria for assessing the selected TMFs

Relevance	Usability
Context—HSE, i.e., to be used by practitioners conducting research rather than full-time researchers Covers complexity of health service Relevant to all disciplines Generating, implementing, exchange, continuum (process + impact)	Easily understood/coherent Practical guidance Important to have examples Research on applying TMF/tested

## Results

### Stage 1: Identifying and evaluating relevant TMFs and initial screening process

The external literature was reviewed by two researchers (KMS and GG). Their role was to identify potentially relevant TMFs. In the course of the review, two recent systematic reviews of TMFs [5, 6] were identified and were used to identify potential TMFs. The Evidence-based Model for the Transfer and Exchange of Research Knowledge (EMTReK) [19] had also been highlighted by one of the project leads prior to screening. Although validation of EMTReK was ongoing at the time of the review (August–December 2019), it had been developed in Ireland and was being used in a health and care context in palliative care. As it was potentially usable in other parts of healthcare, it was decided that this TMF would be included in the review.

**Fig. 2:**
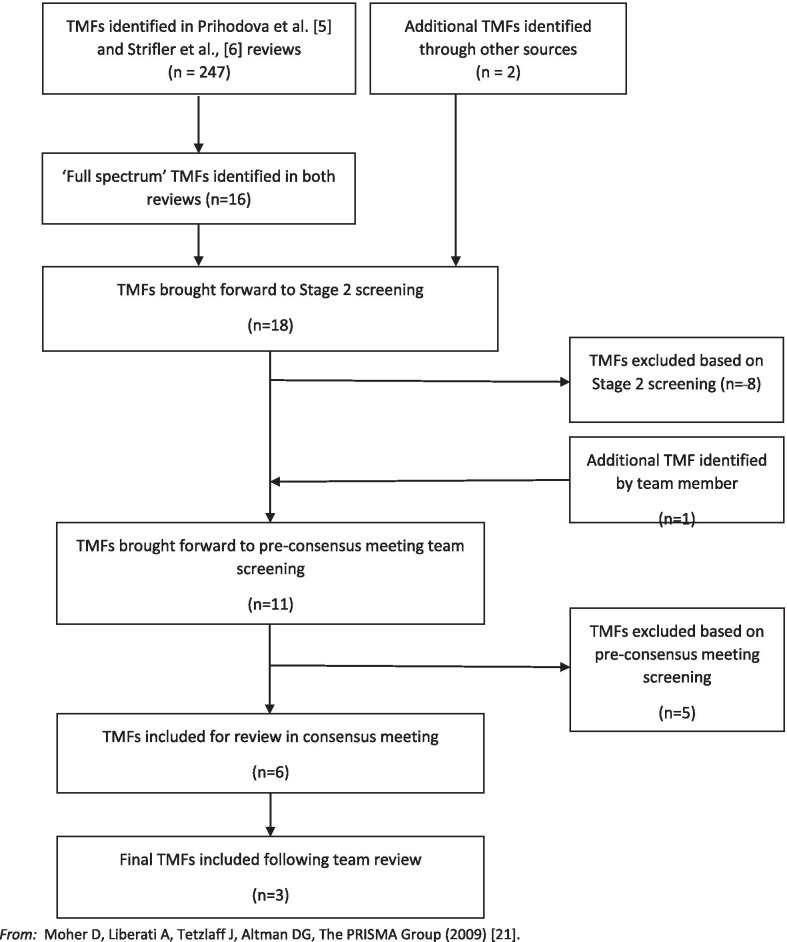
PRISMA [Preferred Reporting Items for Systematic Reviews and Meta-Analyses] diagram. From: Moher D, Liberati A, Tetzlaff J, Altman DG, The PRISMA Group (2009) [21]

In total, 247 TMFs were identified across both systematic reviews (see Fig. 2). The choice of TMFs for screening from the 247 TMFs in both reviews [5, 6] was based on the following two criteria:

- TMFs were identified in both reviews, i.e., overlapped (*N* = 8); and/or
- TMFs were identified in reviews as “full-spectrum”, i.e., used across all four KT stages of planning/design, implementation, evaluation, and sustainability/scalability [20] (*N* = 16).

Based on these criteria, 16 TMFs were identified; the eight TMFs identified in both reviews were also considered “full-spectrum” TMFs.

During the TMF identification process, a systematic review of the Exploration, Preparation, Implementation, Sustainment (EPIS) framework [9] was also published. It was decided to include EPIS in the review due to its relevance and recency. With the inclusion of the EPIS framework and the EMTReK, there were 18 TMFs for review in the second stage of the process (see Table 4).

**Table 4:** TMFs selected following stages 1 and 2 of the screening process

TMFs selected in stage 1 of the screening process	TMFs selected following stage 2 of the screening process
Action research [24]	CFIR [25]
Consolidated Framework for Implementation Research (CFIR) [25]	Diffusion of innovations [26]
Diffusion of innovations [26]	EMTRek [5]
Evidence-based Model for the Transfer and Exchange of Research Knowledge (EMTReK) [5]	EPIS [9]
Exploration, Preparation, Implementation, Sustainment (EPIS) framework [9]	Iowa model [27]
Iowa model [27]	KTA [28]
Knowledge to action (KTA) [28]	Ottawa [29]
Ottawa model [29]	PARIHS [15]
Plan-Do-Study-Act (PDSA) cycles [30]	PRISM [16]
Precede–Proceed [31]	RE-AIM [32]
Promoting Action on Research Implementation in Health Services (PARIHS) [15]	Sustainability of evidenced-based interventions (EBI) [22]
Practical, Robust Implementation and Sustainability Model (PRISM) [16]	Barwick [23]
RE-AIM [32]	
Self-regulation theory [33]	
Social cognitive theory [34]	
Social marketing framework [35]	
Transtheoretical model [36]	

### Stage 2: Screening of TMFs

Following the initial screening, all remaining TMFs were screened by five reviewers (GG, CH, MM, VM, KM-S). Each TMF was independently screened against the above criteria by at least two reviewers, who each assessed whether to include or exclude the TMF. Where differences arose between reviewers in the assessment of whether to include or exclude, a third reviewer (CH) screened the TMF to reach a final decision. This process resulted in eight TMFs being excluded. During the process, an additional TMF and a KT planning template were brought to our attention by one of the project leads [22, 23]. The sustainability of evidence-based interventions (EBI) TMF [22] was currently being used for an unrelated purpose within HSE and so met the criteria for usability and suitability for use in the Irish health context. It was reviewed by the group and deemed suitable for inclusion in the consensus review process. The Barwick planning template was widely used, and its inclusion was also agreed upon. As a result, 11 TMFs and one KT planning template were brought forward to the consensus review process (See Table 4).

### Stage 3: Consensus review and workshop to identify HSE process using T-CaST tool and discussion

Five members of the project working group returned reviews prior to the consensus meeting. A sixth member returned an incomplete set of reviews, so the data were omitted from the analysis. Scores were calculated as per T-CaST instructions, taking the assessors’ average score and using it to assess the fit of the TMF. The instructions further advised that if multiple team members were completing the T-CaST, consideration should be given to calculating average scores across team members.

There was variation in the scoring across each TMF with the exception of two TMFs which were consistently given low scores (i.e., 15 and under) and could clearly be excluded from the final decision-making process (see Table 5). The two TMFs were:

- Diffusion of innovations
- Sustainability of EBI

**Table 5:** Project working group review

TMF	Score from consensus review	
	Total	Average
CFIR	87	17.4
Diffusion of innovations	46	9.2
EMTRek	93	18.6
EPIS	93	18.6
Iowa	94	18.8
KTA	93	18.6
Ottawa	86	17.2
PARIHS	92	18.4
PRISM	99	19.8
RE-AIM	83	16.6
Sustainability of EBI	62	12.4
Barwick	89	17.8

The variation in scoring made it difficult to set a clear cut-off point that would denote whether a TMF should be included in the consensus process. A total score of 20 or more should have indicated that for the majority of categories on the rating sheet there was a score of 3, indicating “very good fit”, and that the TMF should be included in those identified for the final selection at the consensus workshop. This strategy worked when analysing data from three out of the five raters and identified five TMFs with a score of 20+ for each rater. A fourth rater had two scores of 20+ and scored three TMFs at 19. The fifth rater gave consistently low scores and recommended that four TMFs be included in the final selection, even though the overall scores totalled 13 or 14, and three or more categories on the rating sheet had been given a score of one (1 = moderate fit).

Taking the total score and average score across all reviewers, it was possible to identify six TMFs with a total score of 90 or more or average score of 18 or more (see Table 5). Although the Barwick KT planning template fell just outside the criteria, with a total score of 89, the average score was 17.8, and hence it was included. The three TMFs that had total scores between 83 and 87 (Consolidated Framework for Implementation Research [CFIR], Ottawa, and RE-AIM) were excluded to reduce the number of TMFs for final review at the consensus workshop.

The subgroup agreed that the six TMFs highlighted in Table 5 would be submitted for review at the consensus workshop and that the discussion would also include the use of the Barwick planning template as an additional resource for KT planning.

### Consensus workshop

All 11 members of the project working group were invited to take part in a consensus workshop, with eight attending plus a facilitator. Workshop attendees discussed each TMF in small subgroups of three people, and the groups were asked to review the TMF separately and then, through a process of discussion, reach an agreed assessment of whether to include or exclude according to the above criteria. A facilitated plenary session followed in which each group presented its assessment of the TMFs (see Table 6). This assessment demonstrated there was consensus on the rejection of three frameworks, EPIS, Iowa, and PARIHS [Promoting Action on Research Implementation in Health Services]. Both EPIS and Iowa were felt to be more applicable to quality improvement and implementation projects. The terminology used by EPIS was also seen as unclear and therefore potentially difficult to adopt. PARIHS was considered to be too resource-intensive and complex for a health system to use without the infrastructure to support it. The knowledge-to-action (KTA) framework, EMTReK, and PRISM were viewed as usable, and the whole group discussion focused on whether one of these TMFs was the preferred choice.

**Table 6:** Group analysis of the frameworks

EMTReK	EPIS	Iowa	KTA	PARIHS	PRISM	Barwick planning tool
Good for dissemination for completed research but not for continuum Useful for research design from perspective of dissemination Not useful for implementation Missing prioritization	Widely used in different settings Focuses on implementation stages Has its own jargon Terminology not clear Suitable for quality improvement projects/specific use	Change management style Addresses needs and priorities Good for quality improvement projects Focuses on implementation	Widely used, range of stakeholders Engagement with stakeholders not clear Process—unclear Cycle—all stages Easy to follow but needs guidance on use Specifics missing	Widely used, suitable for clinical settings Resource-intensive—needs facilitators More for implementation Complex	Transferable for different disciplines Contains good components Addresses continuum and sustainability Includes patients/stakeholders Useful for design, dissemination, and implementation Has practical orientation Does not focus on Knowledge Exchange	Useful and practical for researchers Concentrates on KT Focuses on design, not on implementation Great tool but not a TMF

### Decision from consensus workshop

**Fig. 3:**
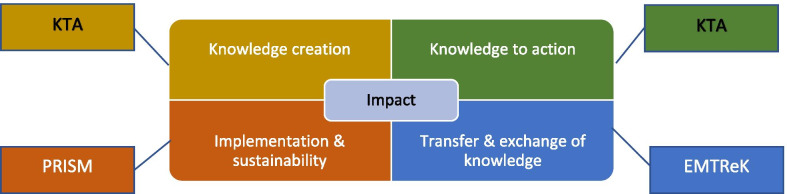
HSE KT process and recommended TMFs

A consensus decision was taken to utilize three TMFs mapped onto the different parts of the KT process (see Fig. 3). This included the KTA framework for the knowledge creation process and KTA cycle, the EMTReK for the transfer and exchange of knowledge, and the PRISM model for implementation and sustainability. The Barwick planning template would be included as an aid to planning.

## Discussion

Previous studies on the use of TMFs in healthcare have demonstrated that they are used partially and variably and that the absence of a TMF to improve the implementation of research findings is a barrier to progress [5, 6, 16, 37]. This underlined the importance of identifying TMFs that could serve as a facilitator to translating research knowledge in HSE. Using the criteria of usability, applicability, and relevance, a limited number of TMFs were assessed as suitable for use by researchers in HSE. However, the T-CaST tool was useful in identifying those that were relevant. Eighteen were identified in stage 1 of the review, reduced to 11 in stage 2, and 6 in the final consensus review. Each of the TMFs in the final selection has specific strengths in its use for HSE (see Table 7). The selected TMFs, namely KTA, EMTReK, and PRISM [16, 19, 28], scored high on usability and acceptability and were validated. Importantly, they appear to be relatively easy to understand for those who are unfamiliar with them, though this will be further tested during a pilot phase.

**Table 7:** Application and strengths of the selected TMFs

KTA	EMTReK	PRISM	Barwick
Knowledge creation Clearly delineated stages of KT Understanding the barriers to implementing research Reaching knowledge users who may benefit from research findings	Developed in the Irish context Focus on the identification of key messages for KT Understanding the barriers to implementing research Reaching knowledge users who may benefit from research findings	Sustaining change Relevance to researchers working on implementation projects Provides guiding questions for users under four domains: Programme (intervention) External environment Implementation and sustainability Recipients	Planning the dissemination of research A tool to guide researchers through the planning process

Previous studies have demonstrated the importance of having a TMF that will be a good fit in a diverse range of health settings and that is flexible enough to work with other frameworks, and this was of significance in the HSE decision to offer an option of three TMFs [37]. The KTA model was also particularly useful in offering clear stages for all elements of KT and will be helpful for researchers engaging in the process without prior experience. It has been widely used in healthcare, with Field et al. [37] identifying 146 studies using some element of the KTA and 10 making it integral to the study implementation.

The strength of EMTReK, in the local context, was its development in Ireland. The TMF was developed in palliative care by the All-Ireland Institute of Hospice and Palliative Care (AIIHPC) between 2013 and 2017. It has been used in five case studies and was shown to be effective in that context [19]. Researchers using this TMF could also benefit from the AIIHPC offer of online learning and resources. Its emphasis on the importance of identifying key messages for dissemination and interactive exchange with stakeholders increases its relevance to other health organizations outside the local context.

The importance of context was emphasized in a number of reviews [5, 9, 37, 38], including the importance of understanding the interaction of context, organization, and stakeholders. Leadership and interaction between senior leaders and knowledge users and collaboration between researchers and decision-makers are issues that were identified as important in other studies [7, 38] and in a survey of HSE researchers [10], and were considered in developing the process for KT. The PRISM model has specific strengths for the implementation of research and those working on implementation projects. The four domains of PRISM also provide a useful guide to implementation and sustainability. Although PRISM is not as widely used as the KTA model [5], the inclusion of factors relating to context and stakeholder engagement, including patients, indicated it could have applicability in the HSE context. The Barwick tool is widely used across health and other sectors. Its value to HSE lies in its application as an aid to planning dissemination of research.

There are over 240 TMFs that may be applicable to health services research [5, 6]. However, many of these, as identified by the two systematic reviews, have not been fully tested or lack evidence of their efficacy [5, 6]. The volume of TMFs identified by our research team made it challenging to identify only one TMF that would be suitable to recommend to all researchers within the HSE. The outcome of the consensus exercise was to offer a menu approach so that researchers might select the TMF that was most suitable for the stage of KT they were working through. Each TMF was felt to have specific merits that warranted its inclusion. However, usability and relevance to the health and care and local (Irish) context were the key criteria in selection. It is clear from this study that for KT to be a core element of health services research, the ability of researchers to operationalize a TMF needs to be considered. The project leads would need to translate and communicate the KT approach to researchers and health service leaders, so the TMFs selected would needed to be understandable. Previous studies support the need for resources, training, and guidance for researchers to enable them to navigate the KT process [5, 7, 37–39].

### Strengths and limitations of the study

We took a consensus approach at all stages, and each part of the process was debated and agreed by a project working group. It was important to draw on the experience and expertise of the members of the project working group, who brought different perspectives and practical knowledge. The importance of spending time at the outset to agree to a shared language and terminology was reinforced at different stages and was particularly important in developing the guidance. However, the study methodology had some limitations. The T-CaST is a recognized and recommended approach, and the approach to the usage of the tool by the project team involving piloting, testing, and reviewing was thorough. The validity of T-CaST is recognized, and it has been recommended for use as an appropriate tool, for example, by Esmail [20], and Damschroder [25]. The adaptation of the T-CaST for local purposes may have compromised the integrity of the tool, although Birken et al. [2] note that users may utilize only the criteria from the tool that are most relevant. While there has been no formal assessment of the reliability of the T-CaST to date, the approach to the development of the T-CaST [2] reflects a comprehensive approach to tool development that included multiple stakeholders to improve reliability and processes to maximize validity.

The addition of three TMFs and a planning tool during stages 1 and 2 was outside the criteria set at the beginning of the review process and was based on local intelligence rather than systematic review in two instances, the EMTReK and sustainability of EBI. Therefore, it is possible that other TMFs which were not included in the reviews or were not familiar to the research team may have been missed. The number of project team members who completed reviews during the consensus process was lower than hoped for. However, more members were able to engage during the workshop, which increases confidence in our findings, particularly given the multidisciplinary backgrounds of workshop members.

## Conclusions

To increase the impact of research in health service organizations, there is a need to build capacity and skills for KT. KT has to be timely and targeted throughout a research study and must take account of the context, whether that is organizational, social, economic, or cultural. Many TMFs focus on individual change and do not consider the system-level change that is frequently a characteristic of health services research. Organizational readiness to create a research culture where knowledge is valued and shared is a key ingredient for successful implementation.

The intended guidance for HSE researchers will provide information that will support them in considering how their research findings can reach the knowledge users who may benefit from them. The tools within the guidance will help researchers in planning how to disseminate their research and reach those who need to know about their findings. The next phase of development for the HSE approach to KT has been to develop a decision support tool to help researchers select the TMF that is most appropriate to their study. Future plans include seeking the views of research active staff on methods of dissemination and KT and piloting the guidance in a range of projects at different stages of the dissemination process. Following the pilot, the guidance will be reviewed, further developed, and rolled out across the organization. Integration of this into a web-based format will also be explored. The literature points to a lack of evaluation within KT, so it will be important to build that in as part of the review process.

There appears to be a gap at present between the development of the TMFs and their practical application. The implication of this work for the future development of TMFs is to consider issues of usability and applicability and how researchers within health service organizations can be supported to translate and mobilize their research into practice.

## Data Availability

Data are available from the authors.

## Abbreviations

CFIR: Consolidated Framework for Implementation Research
EBI: Evidence-based interventions
EMTReK: Evidence-based Model for the Transfer and Exchange of Research Knowledge
EPIS: Exploration, Preparation, Implementation, Sustainment
HSE: Health Service Executive
IKT: Integrated knowledge translation
KTA: Knowledge to action
KT: Knowledge translation
PARIHS: Promoting Action on Research Implementation in Health Services
PRISM: Practical, Robust Implementation and Sustainability Model
PRISMA: Preferred Reporting Items for Systematic Reviews and Meta-Analyses
T-CaST: Theory Comparison and Selection Tool
TMFs: Theories, models, and frameworks

## Footnotes

1 The purpose of the T-CaST tool is as follows:

- Consider the characteristics of the TMFs that are most important for a project.
- Compare potential TMFs to select those with the best fit for a project.
- Identify where more than one TMF might be suitable for use in a project.
- Communicate to stakeholders the reasons that a TMF has been selected.
- Enable transparent reporting of the criteria used to select a TMF.

## References

[1] Terrés A (2019). HSE Action Plan for Health Research 2019–2029.

[2] Birken SA, Rohweder CL, Powell BJ, Shea CM, Scott J, Leeman J, Grewe ME, Kirk MA, Damschroder L, Aldridge WA, Haines ER, Straus S, Presseau J (2018). T-CaST: an implementation theory comparison and selection tool. Implement Sci.

[3] Glasziou P, Chalmers I (2018). Research waste is still a scandal—an essay by Paul Glasziou and Iain Chalmers. BMJ.

[4] Minogue V, Wells B (2018). Adding value, reducing research waste, the role of the NHS research and development management community. Int J Health Gov.

[5] Prihodova L, Guerin S, Tunney C, Kernohan WG (2018). Key components of knowledge transfer and exchange in health services research: findings from a systematic scoping review. J Adv Nurs.

[6] Strifler L, Cardoso R, McGowan J, Cogo E, Nincic V, Khan P, Scott A, Ghassemi M, MacDonald H, Lai Y, Treister V, Tricco A, Straus S (2018). Scoping review identifies significant number of knowledge translation theories, models and frameworks with limited use. J Clin Epidemiol.

[7] Dobbins M, Traynor R, Workentine S, Yousefi-Nooraie R, Yost J (2018). Impact of an organization-wide knowledge translation strategy to support evidence informed public health decision making. BMC Public Health.

[8] McClean R, Graham I, Tetroe J, Volmink J. Translating research into action: an international study of the role of research funders. Health Res Policy Syst. 2018;16(44). 10.1186/s12961-018-0316-y PMC5968540 29793541

[9] Moullin JC, Dickson KS, Stadnick NA, Rakin B, Aarons GA (2019). Systematic review of the exploration, preparation, implementation, sustainment (EPIS) framework. Implement Sci.

[10] Minogue V, Morrissey M. HSE research and development. Research dissemination, knowledge translation, and impact—survey results. Unpublished. 2020. https://hseresearch.ie/research-dissemination-and-translation/.

[11] Royal College of Physicians (2020). Research for all? An analysis of clinical participation in research.

[12] Brownson RC, Kreuter MW, Arrington BA, True WR. Translating scientific discoveries into public health action: how can schools of public health move us forward? Public Health Reports 2006;121. 10.1177/003335490612100118 PMC1497798 16416704

[13] Greenhalgh T, Raftery J, Hanney S, Glover M (2016). Research impact: a narrative review. BMC Med.

[14] European Science Foundation. Impact classification. https://www.esf.org.

[15] Harvey G, Kitson A (2016). PARIHS revisited: from heuristic to integrated framework for the successful implementation of knowledge into practice. Implement Sci.

[16] Feldstein AC, Glasgow RE. A practical, robust implementation and sustainability model (PRISM) for integrating research findings into practice. Joint Commission J Qual Patient Safety. 2008;34(4). 10.1016/s1553-7250(08)34030-6 18468362

[17] Ward V, House A, Hamer S. Knowledge brokering exploring the process of transferring knowledge into action. BMC Health Services Res. 2009. 10.1186/1472-6963-9-12 PMC2632997 19149888

[18] Grimshaw JH, Eccles MP, Lavis JN, Hill SJ, Squires JE. Knowledge translation of research findings. Implement Sci. 7(50). 10.1186/1748-5908-7-50 PMC3462671 22651257

[19] Payne C, Brown MJ, Guerin S, Kernohan WG (2019). EMTReK: an evidence-based model for the transfer & exchange of research knowledge—five case studies in palliative care. SAGE Open Nursing.

[20] Esmail R, Hanson HM, Holroyd-Leduc J, Brown S, Strifler L, Straus SE, Niven DJ, Clement FM (2020). A scoping review of full-spectrum knowledge translation theories, models, and frameworks. Implement Sci.

[21] Moher D, Liberati A, Tetzlaff J, Altman DG (2009). Preferred reporting items for systematic reviews and meta-analyses: the PRISMA statement. PLoS Med.

[22] Shelton RC, Rhoades-Cooper B, Wiltsey-Stirman S (2018). The sustainability of evidence-based interventions and practices in public health and health care. Annu Rev Public Health.

[23] Barwick M (2008). Knowledge translation planning template.

[24] Abad-Corpa E, Cabrero-Garcia J, Delgado-Hito P, Carrillo-Alcaraz A, Meseguer-Liza C, Martinez-Corbalan JT (2012). Effectiveness of participatory-action-research to put in practice evidence at a nursing onco-haematology unit. Rev Lat Am Enfermagem.

[25] Damschroder LJ, Aron DC, Keith RE, Kirsh SR, Alexander JA, Lowery JC (2009). Fostering implementation of health services research findings into practice: a consolidated framework for advancing implementation science. Implement Sci.

[26] Abed J, Reilley B, Butler MO, Kean T, Wong F, Hohman K (2000). Comprehensive cancer control initiative of the centers for disease control and prevention: an example of participatory innovation diffusion. J Public Health Manag Pract.

[27] Titler MG, Kleiber C, Steelman VJ, Rakel BA, Budreau G, Everett LQ, Goode CJ (2001). The Iowa Model of evidence-based practice to promote quality care. Crit Care Nursing Clin North Am.

[28] Graham I, Logan J, Harrison M, Straus S, Tetroe J, Caswell W, Robinson N (2006). Lost in knowledge translation: time for a map?. J Contin Educ Health Prof.

[29] Schroy PC, Emmons K, Peters E, Glick JT, Robinson PA, Lydotes MA, Mylvanaman S, Evans S, Chaisson C, Pignone M (2011). The impact of a novel computer-based decision aid on shared decision making for colorectal cancer screening: a randomized trial. Med Decision Making.

[30] Agurto I, Sandoval J, De La Rosa M, Guardado ME (2006). Improving cervical cancer prevention in a developing country. Int J Qual Health Care: J Int Soc Qual Health Care/ISQua.

[31] Haynes RB, Hayward RS, Lomas J. Revisiting precede-proceed: a leading model for ecological and ethical health promotion. Health Educ J. 2015.

[32] Glasgow RE, Vogt TM, Boles SM (1999). Evaluating the public health impact of health promotion interventions: the RE-AIM framework. Am J Public Health.

[33] Burnette JL, O’Boyle EH, VanEpps EM, Pollock JM, Finkel EJ. 2013. Mind-sets matter: a meta-analytic review of implicit theories and self-regulation. Psychol Bull. 2013;139. 10.1037/a0029531 22866678

[34] Bandura A (2001). Social cognitive theory: an agentic perspective. Annu Rev Psychol.

[35] Berkowitz JM, Huhman M, Heitzler CD, Potter LD, Nolin MJ, Banspach SW (2008). Overview of formative, process, and outcome evaluation methods used in the VERB™ campaign. Am J Prev Med.

[36] Adams MA, Norman GJ, Hovell MF, Sallis JF, Patrick K (2009). Reconceptualizing decisional balance in an adolescent sun protection intervention: mediating effects and theoretical interpretations. Health Psychol: Off J Division Health Psychol, Am Psychol Assoc.

[37] Field B, Booth A, Ilott I, Gerrish K (2014). Using the Knowledge to Action Framework in practice: a citation analysis and systematic review. Implement Sci.

[38] Fitzgerald L, Ferlie E, Wood M, Hawkins C. Interlocking interactions, the diffusion of innovations in health care. Human Relations. 2002;55:1429–1449. Tavistock Institute.

[39] Canadian Institutes of Health Research*.* 2010. About knowledge translation [Internet] Ottawa: The Institutes; http://www.cihr-irsc.gc.ca/e/29418.html.

